# Patient-derived glioblastoma cultures preserve respiration phenotypes during ex vivo maintenance and show sex-associated differences in migration

**DOI:** 10.1186/s40478-026-02349-0

**Published:** 2026-06-18

**Authors:** Veronika Matschke, Philip Glover, Robert Lucaciu, David Pickmann, Martin Scholz, Carsten Theiss, Daniel Hoffmann, Johann Matschke

**Affiliations:** 1 Department of Cytology, Institute of Anatomy, Ruhr-University Bochum, Universitätsstr. 150, Building MA 5/52, 44801 Bochum, Germany; 2 International Graduate School of Neuroscience (IGSN), Ruhr-University Bochum, 44801 Bochum, Germany; 3 Department of Neurosurgery, Academic Teaching Hospital of University Duisburg-Essen, Sana Kliniken Duisburg, 47055 Duisburg, Germany; 4 Bioinformatics and Computational Biophysics, University of Duisburg-Essen, 45117 Essen, Germany; 5 Institute of Cell Biology (Cancer Research), University Hospital Essen, University of Duisburg-Essen, Virchowstraße 173, 45147 Essen, Germany; 6 German Cancer Consortium (DKTK) Partner Site Essen a Partnership Between DKFZ and University Hospital, Essen, Germany

**Keywords:** Glioblastoma, Metabolic heterogeneity, Mitochondrial respiration, Patient-derived models, Tumor invasion, Sex differences

## Abstract

Glioblastoma (GBM) is a highly heterogeneous, invasive brain tumor with profound metabolic plasticity. Patient-derived cultures provide valuable preclinical models, yet the stability and clinical relevance of their metabolic phenotypes during ex vivo maintenance remain unclear. Here, we performed longitudinal functional profiling of mitochondrial respiration, substrate utilization, and migration in primary patient-derived GBM cultures at one and five weeks ex vivo using Seahorse Bioanalyzer assays and wound-healing assays, complemented by exploratory integration with routine clinical parameters (MGMT promoter methylation, p53 expression, sex, and age). Early profiling identified two mitochondrial subgroups distinguished by spare and maximal respiratory capacity, with MGMT promoter methylation observed exclusively in the high-respiration cluster. Over time, most cultures retained their initial phenotype, whereas a subset transitioned from low to high respiration, indicating dynamic adaptation under standardized conditions. Extended culture was accompanied by patient-specific shifts in fuel utilization, including increased glutamine dependency and reduced inter-sample variability in glucose and fatty-acid parameters. Exploratory analyses suggested an association between higher age and lower fatty-acid oxidation capacity and indicated constrained fatty-acid dependency in p53-positive cultures. Migration assays revealed marked inter-sample heterogeneity and a 2–6 times faster gap closure in male-derived than female-derived cultures. Notably, early migratory behavior showed no strong association with OCR-defined metabolic state, and early fuel-flex parameters did not recapitulate OCR cluster structure. Together, these data provide a standardized longitudinal functional benchmark showing that patient-derived GBM cultures preserve intrinsic metabolic heterogeneity while undergoing time-dependent adaptation, and that migration represents an additional, partially independent functional axis. These findings are hypothesis-generating and support stratified, time-sensitive follow-up studies of metabolic and invasive phenotypes in GBM.

**Supplementary Information:**

The online version contains supplementary material available at 10.1186/s40478-026-02349-0.

## Introduction

Glioblastoma multiforme (GBM) is the most common and aggressive primary brain tumor in adults and classified as a WHO grade 4 tumor [1]. Despite advances in surgical techniques, radiotherapy, and chemotherapy, GBM remains an incurable disease with a median survival of only 12–15 months [2]. The high degree of heterogeneity at the cellular, molecular, and metabolic levels, as well as the tumor’s diffuse infiltration into the surrounding brain tissue, contribute significantly to therapy resistance and disease progression [3–5]. While established molecular markers such as IDH1 mutations, ATRX loss, MGMT promoter methylation, Ki67 index, GFAP expression, and nuclear p53 positivity play a crucial role in tumor classification and prognosis [6–11], their relationship with functional metabolic phenotypes in patient-derived models remains insufficiently defined.

In recent years, increasing evidence has highlighted the importance of metabolic reprogramming in GBM pathogenesis [12–17]. Tumor cells exhibit remarkable metabolic plasticity, enabling them to switch between glycolysis and oxidative phosphorylation depending on environmental conditions [18–23]. Mitochondrial metabolism is particularly relevant in this context, as it plays a central role in maintaining tumor cell survival and therapy resistance [23]. While metabolic targeting has been proposed as a therapeutic concept, translating metabolic observations into actionable interventions remains challenging [18, 24, 25], in part because metabolic states are dynamic and context-dependent. A practical prerequisite for any stratified follow-up testing is therefore a standardized functional baseline describing the range, stability, and drift of metabolic phenotypes in patient-derived GBM cultures under defined conditions.

Patient-derived primary glioblastoma cell cultures provide an important tool for studying tumor biology and for functional profiling and preclinical hypothesis generation, as they more closely retain the genetic and phenotypic properties of the parental tumor compared to established cell lines such as U87MG [26–28]. However, a major challenge in translational research is the stability of these primary cultures over time [26]. Previous studies have shown that in vitro culturing conditions can alter cellular properties, including genetic integrity, proliferation rates, and metabolic phenotypes. The choice of culture medium is particularly critical, as serum-containing media favor cell survival but promote differentiation, whereas serum-free conditions supplemented with specific growth factors better preserve stem-like properties [26, 29–31]. These factors must be carefully considered when using patient-derived models to study potential therapeutic approaches, as an altered metabolic profile during long-term culture could compromise the translational relevance of in vitro findings. Despite their broad use, systematic longitudinal functional assessments of mitochondrial respiration phenotypes and substrate utilization in patient-derived GBM cultures are still limited. This uncertainty complicates experimental design and interpretation, particularly when metabolic readouts are compared across laboratories, passages, or time in culture.

In this study, we established patient-derived glioblastoma cultures and performed longitudinal functional profiling of mitochondrial respiration, substrate utilization, and early migratory behavior under standardized ex vivo conditions. By comparing early (one week) and later (five weeks) time points after culture establishment, we aimed to quantify the stability, adaptation, and drift of metabolic phenotypes that are routinely used in preclinical GBM research.

Rather than proposing new molecular mechanisms, our objective was to provide a standardized functional benchmark and practical classification framework for mitochondrial respiration phenotypes in patient-derived GBM cultures, and to assess how these phenotypes relate to routine clinicopathological annotations in a hypothesis-generating manner. Moreover, we examined whether early migratory behavior aligns with metabolic state or represents an independent functional dimension of inter-patient heterogeneity.

By integrating longitudinal metabolic profiling with clinical annotation and migration assays, this work addresses a critical gap in model validation and experimental design, and provides a functional reference for time-sensitive, stratified follow-up studies in glioblastoma.

## Materials and methods

### Human subjects

This study was approved by the Ethics Committee of the North Rhine Medical Association (§15, ref. 2,019,033, February 25 2019). Eligible participants were adults (≥ 18 years) with suspected WHO grade 4 glioblastoma, Karnofsky ≥ 70%, and normal laboratory and organ function. Exclusion criteria included severe comorbidities, immunodeficiency, autoimmune or infectious diseases (HIV, Hepatitis B/C). 14 tumors (8 female, 6 male; 40–81 years; Table 1) yielded stable patient-derived cultures and were included in the analyses. All patient data remain the property of Sana Kliniken Duisburg. Samples were collected during routine glioblastoma resection with 5-aminolevulinic acid (5-ALA, 20 mg/kg; MedicalORDERpharma, Bochum, Ch.B. RD250116-0002) guidance and processed within 1 h. Clinicopathological annotations (IDH1-R132H, ATRX, GFAP, Ki67, p53 immunoreactivity, MGMT promoter methylation) were obtained from routine neuropathological diagnostics of the primary tumor specimens. No additional genomic profiling of the derived cultures was performed in this study, precluding reliable assignment to established GBM molecular subtypes. MGMT promoter methylation status refers to the primary tumor specimen and was not re-assessed after ex vivo culture.

**Table 1:** Clinical and experimental characteristics of patient-derived glioblastoma samples

Sample No	Sex	Age	GBM localization	GFAP-content (%)	IDH1-R132H mutation	ATRX-loss	Ki67 Prolife-ration-index (%)	Nuclear p53 Positivity (%)	MGMT Promoter methy-lation	Mito early only	Mito early & late	Fuel-flex early only	Fuel-flex early & late	Migration ASSAY
011	m	50	Left frontal	70	wildtype	wildtype	15	1	Methylated	✔		✔		✔
012	f	63	Left occipital	90	wildtype	wildtype	15	5	Methylated	✔		✔		✔
013	f	75	Right temporal	80	wildtype	wildtype	15	< 3	Unmethylated	✔		✔		✔
014	f	70	Left temporal	90	wildtype	wildtype	20	25	Unmethylated	✔	✔	✔		✔
015	m	54	Left frontal	80	wildtype	wildtype	10	3	Unmethylated	✔	✔	✔	✔	✔
016	m	74	Bilateral frontal	> 90	wildtype	wildtype	30	> 90	Methylated	✔	✔	✔	✔	✔
017	f	53	Bilateral parietal	90	wildtype	wildtype	30	3	Unmethylated	✔	✔			
018	f	40	Right parieto-occipital	50	wildtype	wildtype	20	60	Unmethylated	✔	✔	✔	✔	✔
019	m	68	Left temporal	> 90	wildtype	wildtype	10	< 5	Unmethylated	✔	✔	✔	✔	✔
029	f	61	Right frontal	60	wildtype	wildtype	20	5	Methylated	✔		✔		✔
031	f	77	Left frontal	80	wildtype	wildtype	15	30	Unmethylated	✔		✔		
032	f	68	Left frontotemporal	80	wildtype	wildtype	20	5	Unmethylated	✔	✔	✔	✔	
035	m	81	Left parietal	20	wildtype	wildtype	40	3	Methylated	✔	✔	✔	✔	✔
036	m	45	Right frontal	70	wildtype	wildtype	40	0	Unmethylated	✔	✔	✔	✔	✔

Summary of patient demographics, tumor localization, and molecular markers, including GFAP expression, IDH1-R132H mutation status, ATRX loss, Ki67 proliferation index, nuclear p53 positivity, and MGMT promoter methylation. The table also indicates which samples were used for Seahorse Bioanalyzer analyses (early only or both early and late) and migration assays

### Isolation and cultivation of primary tumor cells

For isolation of primary tumor cells, tissue was washed three times with 5 mL cold PBS. During processing, macroscopically necrotic regions and blood clots were avoided whenever possible to enrich viable tumor material for culture establishment and downstream functional assays. The tissue was minced until a milky suspension formed, followed by enzymatic digestion in 3 mL pre-warmed 0.25% Trypsin–EDTA (#25,200,056, Thermo Fischer Scientific, Waltham, USA) at 37 °C for 10-15 min. Digestion was terminated by adding 3 mL of DMEM/F-12 medium supplemented with 10% fetal bovine serum (FBS, #16,050-122; Thermo Fisher Scientific, Waltham, USA) and 1% penicillin–streptomycin (#P4333; Sigma-Aldrich, St. Louis, MO, USA). The suspension was gently triturated and centrifuged at 1096 rpm for 10 min at RT. After centrifugation, the cell pellet was resuspended in cultivation medium (5 mL of DMEM/F-12 with 2% FBS and 1% penicillin–streptomycin). A low-serum (2% FBS) condition was used to support short-term viability and assay reproducibility in primary cultures. After filtration through a 100 µm cell strainer (#CLS431752; sigma-Aldrich, St. Louis, MO, USA) a second centrifugation step at 1096 rpm for 5 min at RT was performed. The cell pellet was resuspended in 1 mL cultivation medium. Cell viability was assessed by Trypan Blue (#15,250,061, Thermo Fisher Scientific, Waltham, USA) exclusion. 100,000 cells were seeded in a T25 cell culture flask containing 5 mL cultivation medium. The cells were incubated at 37 °C with 5% CO₂ and 95% humidity. Cultures were routinely monitored and expanded by passaging when reaching ~ 70–80% confluence. Every two days, culture medium was replaced with fresh medium to maintain optimal conditions.

### Metabolic characterization using seahorse bioanalyzer

To assess potential changes in mitochondrial function and metabolic dependencies over time, both the Mitochondrial Stress Test and the Fuel Flex Test were performed on primary glioblastoma cells at two distinct time points: one week ex vivo (early) and five weeks ex vivo (late). One week was selected as the earliest feasible time point after culture establishment and recovery for standardized Seahorse seeding, whereas five weeks captured early adaptation during routine maintenance while limiting prolonged selection. Assays were performed within a consistent daytime window, and samples were randomized across plate positions to minimize systematic bias. This approach allowed us to investigate whether prolonged in vitro cultivation affects mitochondrial respiration and substrate utilization in patient-derived glioblastoma cells.

#### Mitochondrial stress test

Mitochondrial respiration was analyzed according to the manufacturer’s protocol and as previously described [32–35].

Cells were seeded at 10,000 cells per well 24 h prior to the measurement. Before the assay, culture medium was replaced with 180 μl XF DMEM (Agilent Technologies, Santa Clara, CA, USA) supplemented with 1 mM Pyruvate, 2 mM Glutamine, 10 mM Glucose (all Sigma-Aldrich, St. Louis, MO, USA), followed by 1 h-hour CO_2_-free incubation for pH pH equilibration.

Oxygen consumption rates (OCR) and extracellular acidification rate (ECAR) were measured at baseline and after sequential injections of oligomycin (1 μM, Biomol, Hamburg, Germany), carbonyl cyanide 4-(trifluoromethoxy) phenylhydrazone (FCCP, 2 μM, Biomol, Hamburg, Germany), and a mixture of rotenone and antimycin A (0.5 μM, Biomol, Hamburg, Germany), to determine basal respiration, ATP-linked respiration, proton leak, maximal respiration, and spare respiratory capacity. FCCP concentration was determined based on prior titration experiments (0.5–2 µM) in representative cultures, with 2 µM consistently inducing maximal OCR responses. After each assay, cells were fixed with 4% PFA and stained with Hoechst 33,342 (Sigma-Aldrich, St. Louis, MO, USA) for DNA-based normalization using a BioTek Synergy H1 plate reader (Biotek, USA). Baseline OCR prior to inhibitor injections served as an internal within-well reference. OCR remaining after rotenone/antimycin A was interpreted as non-mitochondrial respiration and used to derive mitochondrial parameters according to standard Seahorse conventions. All OCR/ECAR values and derived parameters were normalized to DNA content (Hoechst) to account for well-to-well differences in cell number.

#### Fuel flex test

Metabolic flexibility was assessed using the Seahorse Fuel Flex Test following the manufacturer’s protocol. The assay quantifies dependency, capacity, and flexibility for the three major mitochondrial substrates: glucose (via pyruvate oxidation), glutamine (via glutaminolysis), and fatty acids (via β-oxidation).

Cells were prepared identically to the Mito Stress Test. OCR was measured at baseline and after sequential inhibition of substrate pathways using UK5099 (2 µM, MedChemExpress, Monmouth Junction, NJ, USA), BPTES (3 µM, MedChemExpress, Monmouth Junction, NJ, USA), and Etomoxir (4 µM, MedChemExpress, Monmouth Junction, NJ, USA) as previously described [36]. Fuel dependency, capacity, and flexibility were calculated from OCR changes after single or dual pathway inhibition. Cells were fixed and Hoechst-stained for DNA-based normalization.

### Migration assay of the primary tumor cells

The migratory capacity of isolated primary glioblastoma cells was assessed using a wound healing assay. To ensure reproducibility, specialized cell culture inserts (ibidi Culture-Insert 2 Well in µ-Dish 35 mm; ibidi GmbH, Gräfelfing, Germany) were used. The assay was performed exclusively on primary glioblastoma cultures that had been maintained ex vivo for one week to preserve their in vivo-like phenotype and behavior, thereby minimizing prolonged culture-associated drift and selection effects.

The cell suspension was adjusted to a final concentration of 70,000 cells/mL, and 70 µL of this suspension was carefully applied into both wells of the culture-insert. The cells were incubated at 37 °C with 5% CO_2_ for 24 h. Cell migration was continuously monitored over a 40-h period using time-lapse imaging. Images were acquired every 5 min using a confocal spinning disc microscope (VisiScope Confocal-Cell Explorer, Visitron Systems GmbH, Puchheim, Germany) equipped with a 10× objective (Nikon Achromat Ph1 ADL 10×/0.25; Nikon Instruments Europe BV, Amsterdam, Netherlands).

### Statistical analysis and modeling

#### Barplots

Barplots of data were prepared with GraphPad Prism 10.0 (GraphPad Software, San Diego, CA, USA). Bar heights are means, error bars for three or more replicates are standard errors of the mean.

#### Statistical analysis of mitochondrial parameters

Oxygen consumption rate (OCR) data obtained from the Mito Stress Test were processed and analyzed with R (R Core Team [37]). For visualization, mean OCR values were plotted using the ggplot2 package [38]. OCR parameters were log(x + 1) transformed prior to clustering to focus on order of magnitude changes while retaining cases with zeros.

For each sample *i*, the four transformed OCR values were treated as a four-dimensional vector *x*_*i*_. Each of these vectors was standardized to a vector *z*_*i*_ by subtracting the mean and dividing by the standard deviation of its four components. Pairwise Euclidean distances between standardized vectors *z*_*i*_ were calculated, and hierarchical clustering was performed using ward.D2 linkage (R function *hclust*).

Clustering was initially carried out for early OCR measurements (one week ex vivo). For the subset of nine samples with data available at both early (E) and late (L; five weeks ex vivo) time points, joint clustering of early and late measurements was performed to assess the stability of metabolic subgroups and potential transitions over time.

#### Statistical analysis and modeling of fuel flex data

Fuel Flex Test results were summarized as dependency (d), flexibility (f), and capacity (c) for each of the three major mitochondrial substrates: glucose (Gluc), glutamine (Q), and fatty acids (FA), yielding nine parameters per sample (dGluc, dQ, dFA, fGluc, fQ, fFA, cGluc, cQ, cFA).

To survey relationships between parameters and clinical variables, Spearman rank correlations were calculated for all pairwise combinations. Binary variables (e.g., sex) were numerically encoded for this purpose.

The nine-dimensional parameter vectors were subjected to hierarchical clustering using Euclidean distance and ward.D2 linkage (R function *hclust*). Clustering was performed separately for early (one week ex vivo) measurements and for the combined dataset including both early and late (five weeks ex vivo) measurements.

For the combined early and late dataset, cluster assignment was used to compare the distribution of patient ages between clusters. This was modeled using a Gaussian Bayesian regression with likelihood age ~ Normal(µ_i_, σ), fitted with the *stan_glm* function from the R package rstanarm [39] with weakly informative default priors. Numerical model fit was performed with 4000 steps of Markov Chain Monte Carlo sampling (2000 steps warm-up, 2000 steps sampling). The validity of the fitted model was assessed by visual posterior predictive checks, i.e. comparison of age distribution generated by the model with age distribution of original data. Probability distribution of age difference µ_A_–µ_B_ between clusters was reported as 5–95% credible interval.

#### Statistical analysis and modeling of migration data

For each sample, time intervals displaying approximately linear growth were identified by visual inspection, excluding asymptotic phases at the beginning or end of the trajectories. Linear models were then fitted to these intervals of linear growth to obtain migration slopes. Sample GBM014 represented an exception as gap closure stalled at < 20% after an initial growth phase; in this case, the slope was estimated from the early linear phase only, acknowledging that the model overestimated overall closure.

To compare migration dynamics between male- and female-derived samples, slopes were log-transformed and analyzed with a Bayesian normal model:

y_i_ ~ Normal(µ_s__[i]_, σ), where y_i_ denotes the log-transformed slope of sample i and s[i] indicates the sex of the patient. Weakly informative priors were used (µ ~ Normal(0, 2); σ ~ Exponential(1)), covering biologically meaningful values. The model was implemented in Stan [40] with four Markov chains, each comprising 1000 warm-up iterations and 1000 posterior samples. Model validity was confirmed by posterior predictive checks. The posterior distribution of the male-to-female slope ratio was computed, and the 5% and 95% quantiles of the posterior were used as credible intervals to summarize sex-associated differences of migration speed.

## Results

### Heterogeneous mitochondrial activity in early-stage primary glioblastoma cultures

To assess mitochondrial function in patient-derived glioblastoma cells, we performed the Mitochondrial Stress Test using the Seahorse XFe96 Bioanalyzer after one week ex vivo. For this purpose, the oxygen consumption rate (OCR), a key indicator of mitochondrial respiration and oxidative phosphorylation, was measured in real-time. Additional mitochondrial parameters, such as mitochondrial ATP production and maximal respiration, were assessed through inhibitor-induced changes.

**Fig. 1:**
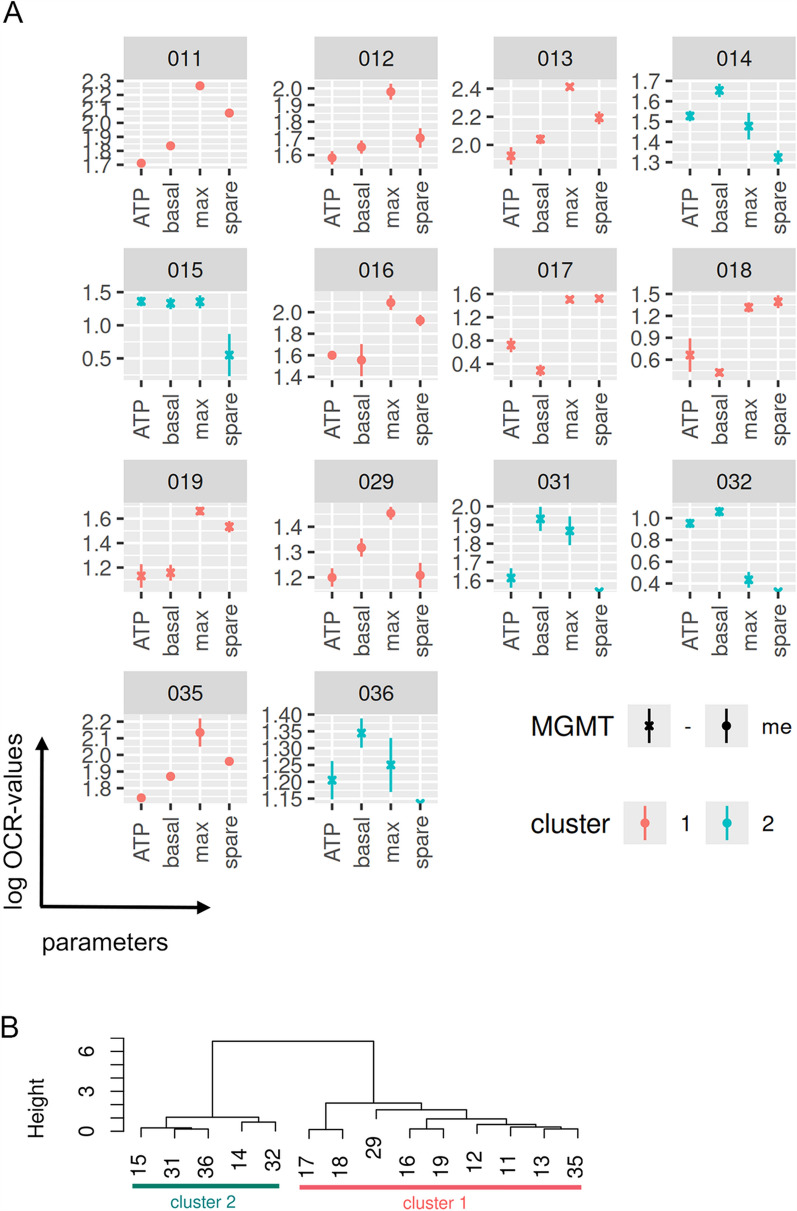
Mitochondrial respiration in patient-derived glioblastoma cells after one week ex vivo. **A** OCR values for mitochondrial ATP production-linked (ATP), basal, maximum, and spare respiration (log-scaled vertical axis) for the 14 samples (sample IDs in panel headers). Cluster 1 in red, cluster 2 in green. Crosses represent neg. MGMT promotor methylation (-), filled circles represent positive MGMT promotor methylation (me). Y-axis scales were adjusted between panels to highlight similarities in curve shapes across samples. Each sample was measured in triplicate. Data represent mean ± SEM. **B** Hierarchically clustering of standardized log(x + 1) transformed OCR values. The leaves of the dendrogram are annotated with the simplified sample IDs (11 = GBM011, etc.). The samples form two clearly distinct clusters

As expected, glioblastoma cultures demonstrated pronounced metabolic heterogeneity at this early time point. Representative measured OCR plots (Supplementary Fig. 1A) illustrate the dynamic and heterogeneous response of glioblastoma cells to sequential injections of mitochondrial inhibitors. Basal and maximal respiration, ATP-linked respiration, and spare respiratory capacity vary widely across samples, ranging from very low to highly elevated rates (Fig. 1A, Supplementary Fig. 1B–P). These findings indicate differences in mitochondrial activity, energy metabolism, and the capacity of individual tumors to adapt to metabolic stress, suggesting a spectrum of bioenergetic phenotypes within the glioblastoma cohort.

Despite the wide heterogeneity in mitochondrial parameters across the cohort, clustering of early OCR data revealed a clear separation into two distinct metabolic clusters (Fig. 1B). Cluster 1 (GBM011, GBM012, GBM013, GBM016, GBM017, GBM018, GBM019, GBM029, GBM035) was characterized by higher maximal respiration and greater spare respiratory capacity, whereas Cluster 2 (GBM014, GBM015, GBM031, GBM032, GBM036) exhibited overall lower OCR values, particularly in the spare respiratory capacity domain (Fig. 1A, Supplementary Fig. 1P). The relative differences between clusters followed a near-proportional up- or down-shift across all OCR parameters (Fig. 1A), suggesting a globally coordinated regulation of oxidative capacity rather than isolated defects in specific respiratory components. The characteristic pattern of the four OCR values becomes evident after standardization of the log-transformed OCR values, i.e. it manifests at different absolute OCR levels. Log transformation was applied to stabilize variance across OCR parameters and to emphasize relative respiration profiles. For instance, in the top left of Fig. 1A, the neighboring samples GBM011, GBM012, and GBM013 show almost identical relative profiles with low ATP-linked respiration, slightly higher basal respiration, dominant maximal respiration, and intermediate spare capacity, while differing in absolute magnitude. This implies that log-scaled OCR values maintain approximately constant ratios within each cluster, reflecting a conserved partitioning of respiratory compartments. As these two clusters emerged under strictly standardized culture conditions, their persistence suggests the existence of two intrinsic mitochondrial phenotypes that are preserved ex vivo.

No significant associations were observed between OCR cluster assignment and the available clinicopathological variables (sex, age, IDH1-R132H status, ATRX, p53 immunoreactivity, Ki67 index, and GFAP). MGMT promoter methylation, assessed in the primary tumor specimen, co-segregated with the high-respiration cluster in this cohort (Fig. 1A). However, this observation did not reach statistical significance (Fisher’s exact test, *p* = 0.086).

The identification of distinct and reproducible OCR-defined clusters under standardized early culture conditions highlights that patient-derived GBM cultures can retain distinct functional respiration phenotypes shortly after establishment. These clusters might reflect pre-existing metabolic states rather than culture-induced artifacts, offering a complementary axis to molecular subclassification. While our data do not address underlying molecular mechanisms, these clusters provide a practical functional stratification framework for longitudinal stability assessments and hypothesis-generating follow-up studies.

### Longitudinal changes of mitochondrial function in patient-derived glioblastoma cultures

Given the known metabolic plasticity and adaptability of glioblastoma cells, we asked whether patient-derived cultures maintain or adapt their mitochondrial respiration phenotypes during ex vivo maintenance. In vitro culture conditions can impose selective pressures and promote functional drift that may affect comparability across time in culture. To investigate these dynamics, we compared mitochondrial function between one week ex vivo and five weeks ex vivo, assessing changes in oxygen consumption rate (OCR), basal respiration, mitochondrial ATP production-linked respiration, maximal respiration, and spare respiratory capacity.

**Fig. 2:**
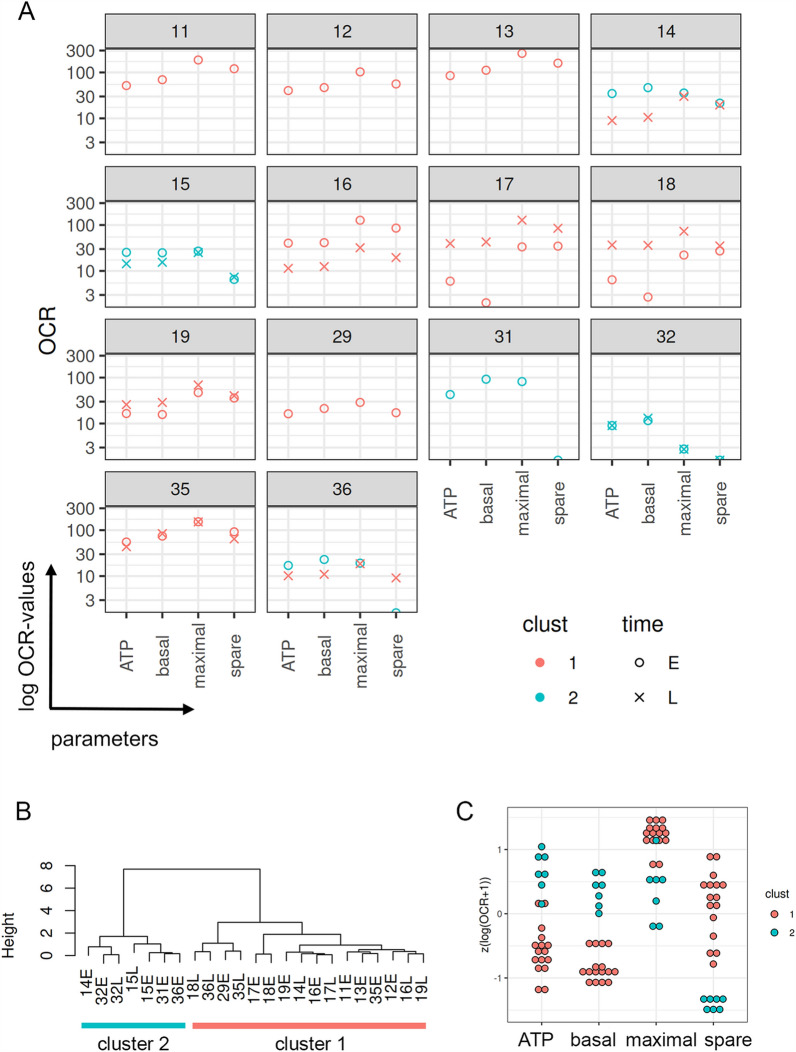
Longitudinal analysis of mitochondrial function in patient-derived glioblastoma cultures. **A** Log-transformed OCR values for ATP, basal, maximum, and spare respiration after one week (E, early) and five weeks (L, late) in culture. Sample IDs in panel headers. Symbols and colors indicate stage (E vs. L) and cluster membership (color). **B** Hierarchically clustering of standardized log(x + 1) transformed OCR vectors z (see Methods). The leaves of the dendrogram are annotated with the sample IDs (sample numbers followed by E for early, L for late). The samples are clearly partitioned into two separate clusters. **C** Clusters 1 and 2 (colors) for all available early and late samples. Vertical axis: standardized log(OCR + 1) values in the four measured categories ATP, basal respiration, maximal respiration, and spare respiration capacity. For samples 11, 12, 13, 29, and 31, only early-stage data were available

OCR measurements revealed patient-specific trajectories of mitochondrial function during cultivation, with some glioblastoma cultures showing increasing and others decreasing respiration over time (Fig. 2A, Supplementary Fig. S2). Hierarchical clustering of the combined early and late OCR data corroborated these observations by confirming the presence of two robust metabolic subgroups (Fig. 2B). Most cultures remained in their cluster after prolonged culture. However, two samples (GBM014, GBM036) transitioned from cluster 2 into cluster 1, whereas no sample moved in the opposite direction. This transition was associated with relative gains in maximal respiration and, for GBM036, recovery of spare respiratory capacity (Fig. 2A). In contrast, cultures such as GBM015 and GBM032 remained in cluster 2, consistent with persistently low spare respiratory capacity.

To facilitate a practical representation of cluster structure across time points, we examined which standardized OCR features best separated the clusters in the combined dataset. In this cohort, basal respiration and spare respiratory capacity captured the dominant separation (Fig. 2C, z-scores), whereas z_ATP_ and z_max respiration_ showed partial overlap. Specifically, samples with z_basal_ < –0.2 or z_spare_ > –1, could be assigned to cluster 1 in this dataset, providing a simple heuristic classification rule for the present cohort.

Together, these data indicate that OCR-defined respiration phenotypes are largely maintained during early ex vivo culture, while a subset of cultures exhibits a directional shift from the low- to the high-respiration cluster. Rather than implying a mechanistic driver, this observation highlights that time in culture can differentially impact respiration capacity across patient-derived models and supports the use of longitudinal OCR phenotyping as a practical framework to monitor stability versus adaptation, thereby capturing differences in mitochondrial adaptive capacity that may be relevant for metabolic stratification strategies..

### Heterogeneous metabolic flexibility and substrate utilization in early-stage primary glioblastoma cells

To characterize mitochondrial substrate utilization in patient-derived glioblastoma cells at one week ex vivo, we performed the Fuel Flex Test using the Seahorse XFe96 Bioanalyzer. This assay quantifies functional flexibility (f), dependency (d), and capacity (c) for oxidation of three major substrate routes: glucose (Gluc, via pyruvate oxidation), glutamine (Q, via glutaminolysis), and fatty acids (FA, via β-oxidation).

**Fig. 3:**
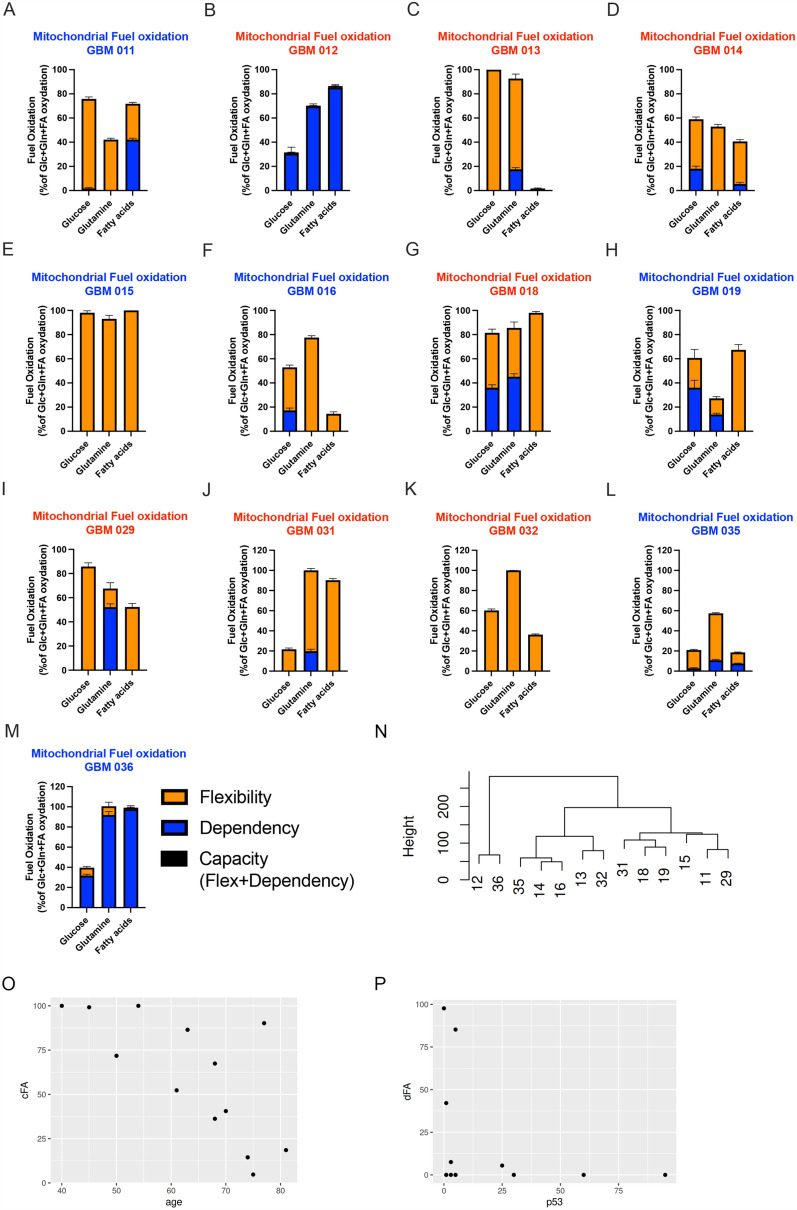
Metabolic substrate flexibility, dependency, and capacity, in patient-derived glioblastoma cells at one week ex vivo. **A**–**M** Fuel Flex Test results for glioblastoma samples assessed at one week ex vivo. Oxygen consumption rates (OCR) were measured under basal conditions and following inhibition of glucose oxidation (Gluc, UK5099), glutamine metabolism (Q, BPTES), and fatty acid β-oxidation (FA, Etomoxir). Dependency (**d**), capacity (**c**) and flexibility (**f**) to oxidize glucose (Gluc), glutamine (Q) and fatty acids (FA) were determined. Sample titles are color-coded according to patient sex: red for female-derived samples and blue for male-derived samples. Each sample was measured in triplicate. Data represent mean ± SEM. **N** Dendrogram for hierarchically clustered data dGluc, dQ, dFA, fGluc, fQ, fFA, cGluc, cQ, cFA for 13 samples (leaf annotations). Apart from the similarity of samples GBM 012 and 036 and the dissimilarity of these two samples to the rest, there are no clear structures. **O** Negative correlation of cFA and age (Spearman rank correlation − 0.7 with non-adjusted p-value 0.0078). Each point corresponds to one sample. **P** Relation between dFA and p53 expression: Samples with detectable p53 show dFA values close to zero, whereas low p53 levels are associated with higher variability in dFA

Across cultures, we observed pronounced inter-patient variability in substrate utilization profiles (Fig. 3A–M). While some glioblastoma cultures exhibited a clear preference or dependency for the utilization of a specific metabolic substrate, others demonstrated a more balanced substrate usage or greater flexibility. For example, GBM011 exhibited a highly adaptable metabolic phenotype, characterized by strong flexibility in switching to oxidation of glucose (74%) and a high capacity for glutamine utilization (74%), indicating robust metabolic plasticity (Fig. 3A). In contrast, GBM012 displayed a high dependency on fatty acid oxidation (85.2%) and low dependency for oxidation of glucose (30.5%), suggesting a lipid-centered metabolic phenotype (Fig. 3B). Meanwhile, GBM013 revealed maximal flexibility and capacity toward glucose utilization (100%) with negligible reliance on fatty acids, indicative of a glycolytic preference (Fig. 3C). Notably, some samples demonstrated restricted flexibility for oxidation of substrates, particularly those with exclusive reliance on one substrate. For example, GBM014 showed moderate dependency and flexibility across all three substrates, reflecting a more plastic metabolic phenotype (Fig. 3D). In contrast, GBM015 exhibited both high dependency (98.1%) and flexibility (93.0%) to oxidize glucose, but minimal use of glutamine or fatty acids, indicating metabolic rigidity (Fig. 3E).

In contrast to the OCR-based respiration phenotypes, hierarchical clustering of Fuel Flex parameters did not yield a robust multi-cluster structure (Fig. 3N). Apart from an outlier pair (GBM012 and GBM036) most cultures did not segregate into discrete groups, suggesting that substrate utilization patterns are more continuous and patient-specific than the OCR-defined respiration phenotypes at this early time point.

Exploratory correlation analyses suggested a negative association between patient age and the capacity for fatty acid oxidation (cFA, ρ = −0.7, non-adjusted *p* = 0.0078), consistent with reports of age-related declines in lipid metabolism (Fig. 3G, Supplementary Fig. 3). In addition, FA dependency values appeared lower and less variable among cultures annotated as p53-positive by immunoreactivity, whereas p53-negative cultures showed a broader range of dFA values (Fig. 3H, Supplementary Fig. 3).

Taken together, these findings underscore the inter-patient variability in mitochondrial substrate utilization in early patient-derived GBM cultures and indicate that fuel usage signatures do not simply recapitulate the OCR cluster structure. This functional dimension may therefore provide complementary information for stratified follow-up testing, while the observed age- and p53-associated patterns warrant validation in larger cohorts and with orthogonal molecular assays.

### Dynamic changes in metabolic fuel dependencies over time in primary glioblastoma cells ex vivo

To determine how glioblastoma cells adapt their metabolic fuel utilization upon cultivation ex vivo over time, we performed the Fuel Flex Test on patient-derived glioblastoma cultures at one week ex vivo and five weeks ex vivo. This assay enabled the longitudinal assessment of mitochondrial substrate usage, specifically glucose, glutamine, and fatty acids, across multiple patient samples.

**Fig. 4:**
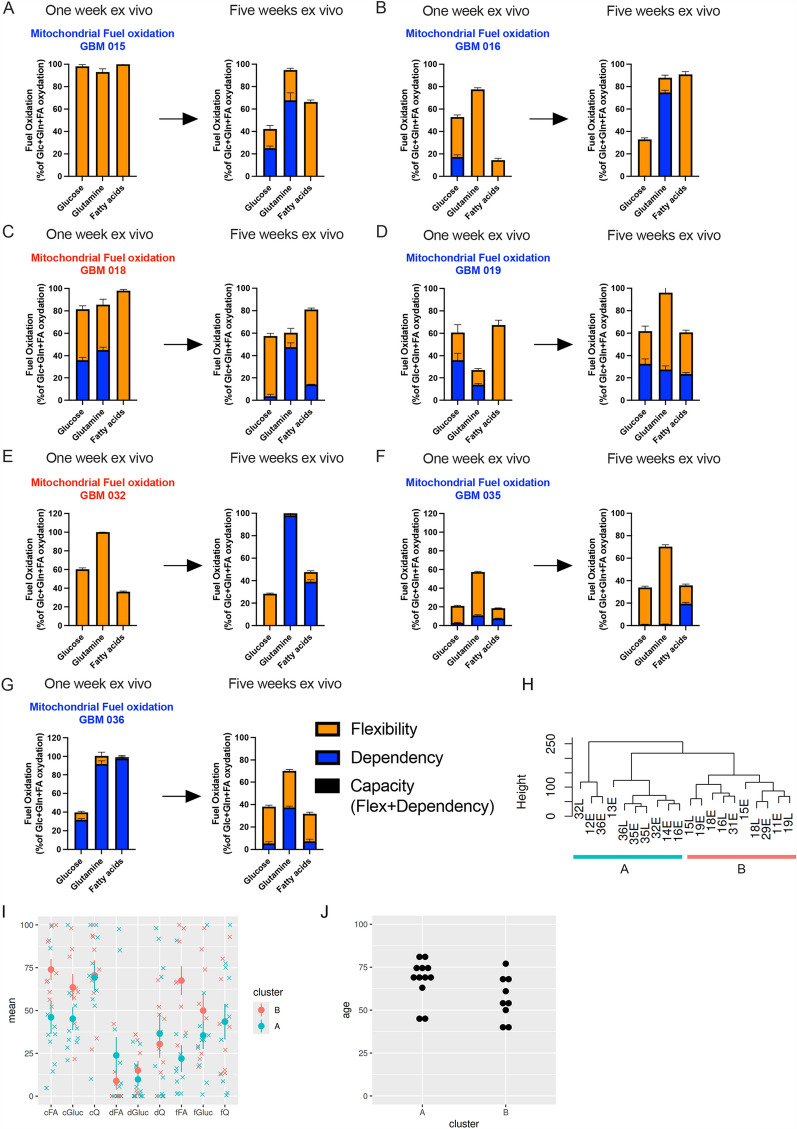
Time-dependent changes in mitochondrial substrate utilization in primary glioblastoma cells. **A**–**G** Fuel Flex Test results for glioblastoma samples assessed at one week ex vivo and five weeks ex vivo. Oxygen consumption rates (OCR) were measured under basal conditions and following inhibition of glucose oxidation (Gluc, UK5099), glutamine metabolism (Q, BPTES), and fatty acid β-oxidation (FA, Etomoxir). Dependency (d), capacity (c) and flexibility (f) to oxidize glucose (Gluc), glutamine (Q) and fatty acids (FA) were determined. Over time, glioblastoma cells displayed an increased reliance on metabolic substrates, with a general shift toward glutamine dependence. Sample titles are red for female-derived samples and blue for male-derived samples. Each sample was measured in triplicate. Data represent mean ± SEM. **H** Hierarchical clustering dendrogram of samples (numbers) for early (“E”) and late (“L”) measurements. Hierarchical clustering of OCR parameters. The cluster containing samples 32, etc. is referred to as cluster A (left side), whereas the cluster containing samples 15, etc. is designated as cluster B. **I** Means of Fuel Flex Test values per cluster (green: cluster A, red: cluster B). Crosses are values per sample and category, point with range is mean per cluster and category ± standard error of the mean. **J** Distribution of ages in clusters A and B

Our experimental analysis revealed considerable inter-patient variability in substrate preferences, underscoring the metabolic heterogeneity of GBM (Fig. 4, Supplementary Fig. 4A). Several cultures demonstrated marked temporal shifts in their fuel dependency patterns. For example, GBM016 shifted from predominant glucose dependency at the early time point to increased glutamine reliance after prolonged cultivation ex vivo, indicating a potential reprogramming toward glutaminolysis (Fig. 4B). GBM032 exhibited a similar trajectory, evolving from an initially balanced substrate utilization profile to a state of strong glutamine dependence after five weeks ex vivo (Fig. 4E). In contrast, GBM035 revealed a shift toward exclusive reliance on fatty acid oxidation (Fig. 4F), while GBM036 displayed a reverse pattern moving from high dependency on all three substrates to a more flexible and less specialized metabolic phenotype after five weeks ex vivo (Fig. 4G). These changes were heterogeneous across cultures, indicating that time in culture does not impose a uniform directional shift on substrate utilization.

Computational integration of early and late Fuel Flex data corroborated this heterogeneity. Hierarchical clustering of all samples suggested two weakly separated metabolic subgroups (clusters A and B), each containing both early and late measurements of the same tumors, indicating that individual cultures largely retained their relative, patient-specific substrate utilization profiles over time (Fig. 4H). In this dataset, cluster A was characterized by lower fatty acid-related parameters (capacity (cFA) and flexibility (fFA)) (Fig. 4I) and was enriched for samples from older patients (Fig. 4J). A Bayesian model estimated that members of cluster A were by a median 11 years older than those of cluster B (90% credible interval for age difference from 1.8 to 20.4 years with median 10.8 years).

Direct comparison of early and late measurements revealed no consistent global shift across the cohort of GBM patient samples but suggested a reduction in variability for several parameters (notably glucose flexibility (fGluc) and fatty acid dependency (dFA)) and an upward trend in glutamine dependency (dQ), as near-zero glutamine usage observed at the early stage was largely absent after five weeks (Fig. 4I).

Taken together, these findings show that mitochondrial fuel utilization in patient-derived GBM cultures is not static: some cultures undergo substantial time-dependent shifts under standardized maintenance, whereas others retain stable, patient-specific signatures. In this cohort, age co-varied with Fuel Flex clustering, with older patients enriched in the group displaying lower fatty acid capacity and flexibility (mean difference ~ 11 years; 90% credible interval 1.8–20.4 years). Although descriptive, this pattern motivates targeted validation and suggests that demographic context and time in culture can influence functional metabolic phenotypes captured by Seahorse assays. These results therefore support time-sensitive, stratified experimental design rather than assuming a single fixed metabolic state.

### Sex-dependent differences in migratory behavior of patient-derived glioblastoma cells

**Fig. 5:**
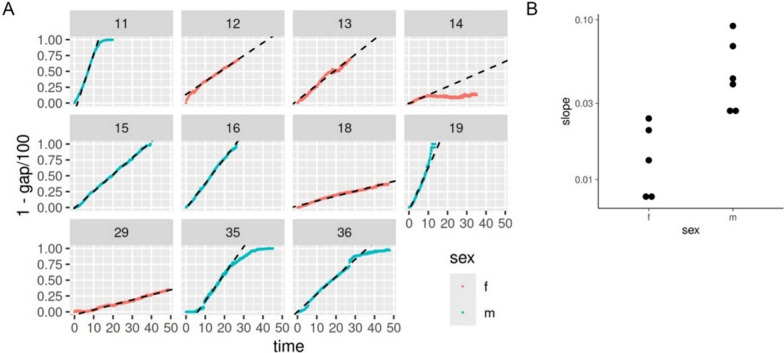
Migration dynamics of patient-derived glioblastoma cells at one week ex vivo. **A** Time-lapse wound healing assay illustrating the gap closure behavior of individual glioblastoma cultures over 35 h. Measured gap closures (red, green) and linear fits (dashed line, black). For the eleven samples (IDs in panel tops) a linear model was fitted to the linear part of the dynamics. Horizontal axis: times in units of hours, vertical axis: degree of gap closure from 0 = gap open, to 1 = gap closed), Colors indicate sex (females red, males green). Fast-migrating cultures such as GBM011 and GBM019 closed the wound area within 12–15 h, whereas slower-migrating cultures (e.g., GBM014, GBM018, GBM029) did not achieve complete closure even after 35 h. **B** Slopes of fitted linear models (dashed lines in A) as function of sex (male m, female f). Vertical axis is log-scaled. These results highlight both the inter-patient heterogeneity and sex-dependent differences in glioblastoma cell motility

To further investigate the invasive potential of patient-derived glioblastoma cells, we performed wound healing assays on primary glioblastoma cultures at one week ex vivo cultivation. The results revealed marked heterogeneity in migration dynamics across individual samples (Fig. 5A). Some glioblastoma cultures, such as GBM011 and GBM019, achieved near-complete gap closure within 12–15 h, indicating high migratory activity. In contrast, other samples such as GBM014, GBM018, and GBM029 displayed markedly slower migration, with the cell-free area remaining incompletely closed even after 40 h. Notably, GBM014 represented an outlier: after an initial growth phase, gap closure stalled at < 20% and remained incomplete throughout the observation period.

Stratification by patient sex revealed a notable pattern in this cohort: glioblastoma cells derived from male patients migrated significantly faster than those from female patients (Fig. 5B, C). Male-derived cultures closed the wound gap more efficiently within the observation period (Fig. 5B), whereas female-derived cultures demonstrated a slower and often incomplete gap closure (Fig. 5C). To quantify this observation, we applied Bayesian modeling to estimate and compare the linear slopes of wound closure over time between male- and female-derived cultures (Fig. 5D, Supplementary Fig. 5). The model validity was confirmed by a posterior predictive check (PPC), and the analysis revealed that the posterior distribution of the male-to-female slope ratio ranged from 1.8 to 6.0 with 90% confidence (Fig. 5E), i.e. the male-derived glioblastoma cell cultures close the experimental gap about two to sixfold faster than the female-derived cultures. This statistically supports a meaningful sex-dependent difference in ex vivo migration speed.

To assess whether migratory behavior reflects metabolic state, we compared early gap-closure slopes with OCR-defined clusters and metabolic parameters. Exploratory analyses revealed no strong association between migration rates and OCR cluster assignment (Supplementary Fig. 3). These findings indicate that early migratory behavior constitutes a functional axis that is largely independent of mitochondrial respiration state and substrate utilization signatures in this dataset.

These findings suggest that patient sex may influence the intrinsic migratory behavior of glioblastoma cultures, even under standardized in vitro conditions. This observed sex-dependent difference, embedded within the broader heterogeneity of glioblastoma, may contribute to differential invasion patterns and therapeutic responses. Future studies in larger, genetically stratified cohorts are needed to validate these observations and to elucidate the underlying molecular mechanisms.

## Discussion

This study provides a longitudinal functional characterization of metabolic heterogeneity and migratory behavior in patient-derived glioblastoma (GBM) cultures under controlled ex vivo conditions, addressing the limited understanding of their stability and temporal adaptation. Specifically, we examined whether mitochondrial function and substrate dependencies are temporally dynamic and whether these experimental findings correlate with clinically accessible tumor markers. In addition, we assessed whether patient sex or other clinical parameters influence the motility-related behavior of patient-derived GBM cultures.

Our integrative analysis of Seahorse-based metabolic profiling revealed pronounced heterogeneity in mitochondrial respiration and substrate utilization among patient-derived GBM cultures, even at an early time point (one week ex vivo). These data are consistent with previous studies reporting substantial variability in oxidative phosphorylation capacity and metabolic plasticity in GBM cells [41–46]. The high diversity observed in mitochondrial oxygen consumption rates (OCR), ATP-linked respiration, and spare respiratory capacity underscores the complex metabolic landscape of GBM, reflecting differences in tumor origin, genetic background, and possibly tumor microenvironmental adaptation [47–49]. Importantly, we identified dynamic metabolic adaptations of patient-derived glioblastoma samples upon prolonged ex vivo cultivation. Some glioblastoma cultures increased their oxidative capacity and substrate dependency during extended ex vivo cultivation, while others showed a decline, without following a consistent or predictable pattern. This aligns with emerging concepts that metabolic reprogramming in glioblastoma is not static but evolves in response to environmental stressors, including nutrient availability and culture conditions [23, 50–53]. A subset of cultures demonstrated shifts toward glutamine dependency or fatty acid oxidation after five weeks ex vivo. Given standardized culture conditions, these observations likely reflect active metabolic adaptation rather than substrate depletion [54–57].

Our data also highlight that ex vivo cultures often preserve intrinsic heterogeneity but are not always metabolically static. Clustering of OCR data revealed two distinct mitochondrial phenotypes that were preserved under standardized ex vivo conditions. The two clusters were characterized by proportional scaling of all OCR parameters within each cluster, but with distinct absolute magnitudes between clusters. This pattern suggests that the separation does not arise from isolated pathway defects, but rather from globally different levels of mitochondrial abundance or respiratory-chain organization. Our interpretation that this proportional scaling reflects variations in mitochondrial abundance and ultrastructure is supported by previous studies showing that both cristae architecture and mitochondrial DNA copy number correlate with oxidative phosphorylation capacity [58–61]. Similar conclusions were reached in a recent glioma study demonstrating that mitochondrial protein density and biomass strongly predict bioenergetic capacity and therapeutic responsiveness [62], further supporting the notion that coordinated mitochondrial content and structure underlie global differences in oxidative capacity. The confinement of MGMT-methylated tumors to the high-OCR cluster suggests co-segregation of MGMT methylation with a respiration-rich state. While we lacked residual material for orthogonal wet-lab validation (e.g., citrate-synthase activity, mtDNA copy number, supercomplex abundance), the preserved coupling ratios and the high goodness-of-fit for simple scaling models argue for stable, cell-intrinsic energetic programs that are maintained ex vivo.

Importantly, these OCR-defined clusters remained stable over time and even allowed for simple classification based on basal respiration and spare capacity, with a subset of tumors transitioning from low- to high-respiration phenotypes but not vice versa, indicating that some tumors can regain respiratory capacity. Notably, transitions between clusters occurred only from low- to high-respiration phenotypes but not in the reverse direction during prolonged cultivation. This directional shift indicates that at least a subset of GBM cultures can regain respiratory capacity under stable culture conditions, consistent with adaptive remodeling rather than stochastic fluctuation. Such recovery of mitochondrial function is consistent with functional plasticity during early ex vivo maintenance and may be relevant for model stability and time-sensitive experimental design. Consistent with this, a tendency was observed for MGMT-methylated tumors to reside in the high-respiration cluster, pointing to a possible link between mitochondrial reserve capacity and clinicopathological tumor annotation.

Longitudinal Fuel Flex analysis revealed more continuous, patient-specific substrate usage patterns rather than discrete clusters, showing that while overall profiles remained patient-specific, subtle cohort-level shifts occurred, including reduced variability in glucose flexibility and fatty acid dependency and an upward trend in glutamine utilization. These findings raise important considerations for the stability and translational relevance of primary GBM cultures.

Short-term cultures may better reflect in vivo tumor metabolism, whereas extended cultivation may introduce metabolic drift [26, 63, 64]. In this context, it is important to consider that in vitro culture conditions, particularly the use of serum-containing media, may promote partial differentiation and alter stemness-associated properties in patient-derived GBM cultures. Such shifts in cellular state have been reported to influence metabolic phenotypes [65, 66] and could therefore contribute to the temporal adaptations observed in our dataset. While our exploratory qPCR analyses (data not shown) did not indicate a uniform and consistent loss of stem/progenitor-associated marker expression across cultures, they do not exclude more subtle or culture-specific changes in cellular state. Accordingly, the observed metabolic heterogeneity and adaptation should be interpreted within the context of the applied culture system, in which metabolic and phenotypic plasticity may be partially uncoupled from classical stemness-associated features. More broadly, alternative systems such as organoid cultures may better preserve patient-specific traits but remain limited in capturing the microenvironmental complexity that shapes metabolic phenotypes [67, 68]. Overall, these results underscore the importance of rigorously defined culture conditions and early measurement time points, as media composition and culture duration can substantially alter GBM phenotypes and drug responses [26].

Integrative correlation analyses revealed only a few clinical associations with metabolic or migratory phenotypes. Most classical markers, including IDH1 mutation status, ATRX loss, GFAP expression, Ki67 proliferation index, and p53 positivity, showed no robust correlations in this dataset. However, exploratory analyses highlighted several noteworthy trends: MGMT promoter methylation, observed exclusively in the high-respiration cluster, further supported a link between mitochondrial reserve capacity and metabolic phenotype. Exploratory analyses linked patient age and p53 expression to fatty acid metabolism (see below for mechanistic discussion). In the longitudinal Fuel Flex dataset, clustering also separated samples by age, with older patients enriched in the fatty acid-associated cluster. Together, these observations suggest that patient age, MGMT status, and p53 expression may influence specific metabolic phenotypes, although validation in larger cohorts is needed. These findings are in line with prior work suggesting that aggressive tumors exhibit increased metabolic flexibility and a departure from glycolytic reliance [69–71].

Although functional and metabolic properties vary substantially across healthy brain regions, including higher resting-state metabolic activity in fronto-parietal association areas compared to primary sensory cortices [72, 73], our exploratory analyses did not indicate that macroscopic tumor localization explains the metabolic clustering or migration phenotypes observed in our cohort (data not shown). Given the limited cohort size and considerable variability of measurements, we cannot detect smaller effects. So, we can neither confirm nor exclude that regional anatomical context contributes to ex vivo metabolic and migratory behavior. To detect such contributions would require larger anatomically stratified datasets integrating metabolic profiling with imaging-based readouts of local perfusion and connectivity.

Beyond association, recent studies suggest a mechanistic link between epigenetics and lipid-mitochondrial coupling in GBM: multi-omics and label-free imaging indicate distinct lipid-metabolic states in MGMT-methylated versus unmethylated tumors, including altered acyl-chain composition and reduced lipid droplet content in unmethylated GBM [74, 75]. These patterns, together with our finding that MGMT methylation maps to the high-respiration OCR cluster, are compatible with a model in which MGMT status may co-stratify with mitochondrial reserve and lipid-related metabolic states. Mechanistically, p53 has also emerged as a central regulator of lipid metabolism, promoting fatty-acid oxidation, including peroxisomal β-oxidation, and thereby constraining lipid-utilization plasticity [76–78]. This is consistent with our observation that p53-positive samples showed lower and less variable fatty-acid dependency in this dataset. Moreover, metabolomic profiling of pediatric versus adult gliomas has suggested stronger FAO reliance in adult tumors, supporting our age-associated Fuel Flex signatures [79]. Together, these data strengthen the view that MGMT status, p53 expression, and patient age may define distinct mitochondrial-lipid axes in GBM biology. From a therapeutic perspective, fatty acid oxidation has been proposed as a hub of metabolic plasticity in GBM, and combined inhibition strategies (e.g., glycolysis plus FAO blockade) have shown synergistic effects in preclinical models [43, 80]. Thus, lipid-targeted interventions, including statins, are attractive candidates for stratified therapeutic approaches, potentially guided by MGMT methylation or age. While not tested here, these concepts provide a rationale for stratified follow-up experiments motivated by functional phenotyping (e.g., age- or MGMT-annotated subgroups).

To extend metabolic profiling towards functional behavior, we assessed migratory behavior of patient-derived cultures. Migration represents a key feature of GBM aggressiveness and invasion and is a major determinant of disease progression and therapeutic resistance [81, 82]. Given emerging evidence that metabolic adaptations can influence cellular processes such as motility and invasion, we aimed to explore whether inter-patient differences in metabolic substrate utilization and flexibility are associated with differences in migratory capacity. Consistent with the aggressive infiltrative nature of GBM, most cultures demonstrated substantial migratory activity, but with distinct differences in speed and completeness of gap closure. Notably, glioblastoma samples derived from male patients migrated significantly faster than those from female patients, with Bayesian modeling confirming a 1.8 to sixfold faster closure of an experimental gap in male-derived than female-derived samples. This sex-associated effect aligns with recent evidence suggesting sex-specific molecular profiles and therapeutic responses in GBM [83–85]. Recent integrative studies also highlight sex-dependent transcriptional signatures and divergent clinical trajectories, underscoring the importance of considering sex as a biological variable in GBM research [83, 86]. The underlying mechanisms remain unclear, but sex hormones, differential gene expression, and immune modulation have been proposed as contributing factors [83, 87, 88].

Clinically, female GBM patients often show a higher frequency of MGMT promoter methylation [86, 89] and slightly better overall survival [87], both consistent with reduced invasive potential and enhanced therapy responsiveness. Although imaging and volumetric studies report heterogeneous findings [90], the tendency toward slower ex vivo migration in female-derived cultures may reflect a biological correlate of these clinical patterns. Together, these results support the view that sex-specific intrinsic programs, spanning mitochondrial, metabolic, and transcriptional regulation, contribute to functional heterogeneity in glioblastoma. Beyond sex, no consistent clinical predictors of migration were identified in our dataset. Although metabolic plasticity and migration are both critical features of GBM biology, our findings indicate that mitochondrial respiration, substrate utilization, and migratory behavior represent partially independent and context-dependent functional dimensions of GBM heterogeneity under the investigated conditions. Notably, temporal changes in metabolic fuel utilization were not consistently reflected in functional phenotypic endpoints, indicating that metabolic adaptation and cellular behavior are not uniformly coupled in patient-derived GBM cultures under these conditions. However, proliferation analyses performed in a subset of cultures under matching experimental conditions indicated only minimal proliferative activity during the relevant time frame. In addition, time-lapse imaging revealed active and directed cell movement into the wound area, with individual cells migrating out of the cell layer into the gap (representative frames shown in Supplementary Fig. 6). Together, these observations support that wound closure in our experimental setup is predominantly driven by cell motility rather than proliferation.

### Limitations of the study

This study was designed to establish a standardized and reproducible functional framework for longitudinal metabolic and migratory profiling in patient-derived GBM cultures under controlled ex vivo conditions. This focused experimental design enables controlled comparison across samples and time points, but necessarily restricts the analysis to selected functional dimensions and does not capture the full biological complexity of glioblastoma, particularly with regard to molecular and phenotypic characterization.

A key limitation is the restricted molecular and phenotypic characterization of the analyzed cultures. Comprehensive molecular subtype classification and functional assessment of stemness-associated properties were beyond the scope of this study and would require additional dedicated assays and multi-omics profiling. Accordingly, individual cultures could not be reliably assigned to established GBM molecular subtypes such as proneural, classical, or mesenchymal.

Furthermore, the applied culture conditions may influence cellular state. While serum-free conditions are commonly used to preserve stem-like properties in patient-derived GBM cultures [65, 66], our study employed adherent, serum-containing conditions to enable robust and reproducible longitudinal metabolic profiling across multiple patient-derived samples. As a consequence, culture-dependent shifts in differentiation state cannot be excluded and may contribute to the observed metabolic adaptations. Therefore, the reported metabolic phenotypes described here should be interpreted within the context of the defined experimental system.

To partially address this aspect, exploratory qPCR analyses were performed in a subset of cultures across early and late time points of lineage-, stemness- and subtype-associated markers (data not shown). These analyses revealed heterogeneous, marker-specific expression patterns without evidence of a uniform loss of stem/progenitor-associated markers over time. While limited in scope and not replacing functional assays such as tumorsphere formation or limiting dilution analysis, these findings suggest that the observed metabolic phenotypes are not solely driven by a global loss of stemness during short-term ex vivo propagation.

Another limitation is the restricted integration of functional endpoints. Although migration was assessed as a complementary phenotype, no longitudinal analysis of migratory behavior was performed, precluding evaluation of temporal coupling between metabolic adaptation and cell motility. More broadly, the absence of consistent associations between metabolic and functional readouts indicates that these dimensions represent partially independent and context-dependent aspects of GBM biology that are not fully captured by single experimental approaches.

Future studies integrating longitudinal metabolic profiling with extended functional phenotyping, including migration and stemness-associated assays, as well as molecular classification, will be required to better define the relationship between metabolic adaptation, cellular state, and tumor behavior.

## Conclusion

Taken together, our findings highlight the need for precision approaches in GBM research. The pronounced patient-to-patient variability in metabolism and migration underscores the limitations of generalized models and supports stratified, patient-aware experimental frameworks and prioritization of follow-up hypotheses. Moreover, the dynamic nature of GBM metabolism during in vitro culture emphasizes the importance of carefully selecting experimental time points and conditions. Recent integrative studies combining multi-omics with metabolic phenotyping in glioblastoma support this notion and advocate for more comprehensive profiling to identify clinically meaningful subgroups [91–93]. Emerging in vitro models such as tumor organoids or 3D cultures in defined media may improve the preservation of tumor-specific traits, but whether they accurately recapitulate metabolic phenotypes and their dynamics remains unclear [94–96].

This study provides a standardized longitudinal functional benchmark of patient-derived glioblastoma cultures, showing that intrinsic metabolic heterogeneity is preserved early ex vivo but can undergo time-dependent adaptation under standardized maintenance conditions. By combining mitochondrial respiration profiling with fuel utilization readouts and early migration assays, we identify two reproducible OCR-defined respiration phenotypes, patient-specific shifts in substrate dependency, most prominently toward glutamine, and pronounced inter-sample variability in migratory behavior with a consistent sex-associated difference. Importantly, exploratory integration with routine clinical annotations suggests that MGMT status, p53 positivity, age, and sex may co-stratify with specific functional patterns, while these associations remain hypothesis-generating in the absence of causal perturbation or transcriptomic profiling. Together, our results emphasize that mitochondrial respiration state, substrate utilization, and migration represent partially independent functional dimensions of GBM biology that should be considered when designing and interpreting experiments in patient-derived GBM models. These findings motivate time-sensitive, stratified follow-up experiments and underscore the value of functional phenotyping as a practical complement to established clinicopathological markers for evaluating model stability and prioritizing testable hypotheses in glioblastoma biology.

## Supplementary Information

Supplementary Material 1

## Data Availability

All data supporting the findings of this study are included within the manuscript or supplementary information files.
