# Supplementary material for: Patient-derived glioblastoma cultures preserve respiration phenotypes during ex vivo maintenance and show sex-associated differences in migration
Source: Acta Neuropathol Commun. 2026 Jun 18;14:131. doi: 10.1186/s40478-026-02349-0 (PMC13282857; doi:10.1186/s40478-026-02349-0)
Supplement: Supplementary file 1 — Supplementary Material 1 [file 40478_2026_2349_MOESM1_ESM.pdf]

Supplemental Information

Patient-derived glioblastoma cultures preserve respiration phenotypes during *ex vivo* maintenance and show sex-associated differences in migration

Veronika Matschke[1,2#*], Philip Glover[1#], Robert Lucaciu[3], David Pickmann[1], Martin Scholz[3], Carsten Theiss[1,2], Daniel Hoffmann[4], Johann Matschke[5,6*]

[1] Department of Cytology, Institute of Anatomy, Ruhr-University Bochum, 44801 Bochum, Germany

[2] International Graduate School of Neuroscience (IGSN), Ruhr-University Bochum, 44801 Bochum, Germany

[3] Department of Neurosurgery, Sana Kliniken Duisburg, Academic Teaching Hospital of University Duisburg-Essen, 47055 Duisburg, Germany

[4] Bioinformatics and Computational Biophysics, University of Duisburg-Essen, 45117 Essen, Germany

[5] Institute of Cell Biology (Cancer Research), University Hospital Essen, University of Duisburg-Essen, 45147 Essen, Germany

[6] German Cancer Consortium (DKTK) partner site Essen a partnership between DKFZ and University Hospital, Essen, Germany

# These authors contributed equally to this work and share first authorship.

* Corresponding authors: Veronika Matschke, Ruhr University Bochum, Institute of Anatomy, Department of Cytology, Universitätsstr. 150, 44801 Bochum, Building MA 5/52, Phone: +49 234 32 25018, E-Mail: veronika.matschke@rub.de, ORCID 0000-0001-9717-4485;

Johann Matschke, University of Duisburg-Essen, University Hospital Essen, Institute of Cell Biology (Cancer Research), Virchowstraße 173, 45147 Essen, Phone: +49 201 7234234, E-Mail: johann.matschke@uk-essen.de, ORCID 0000-0003-4878-8741

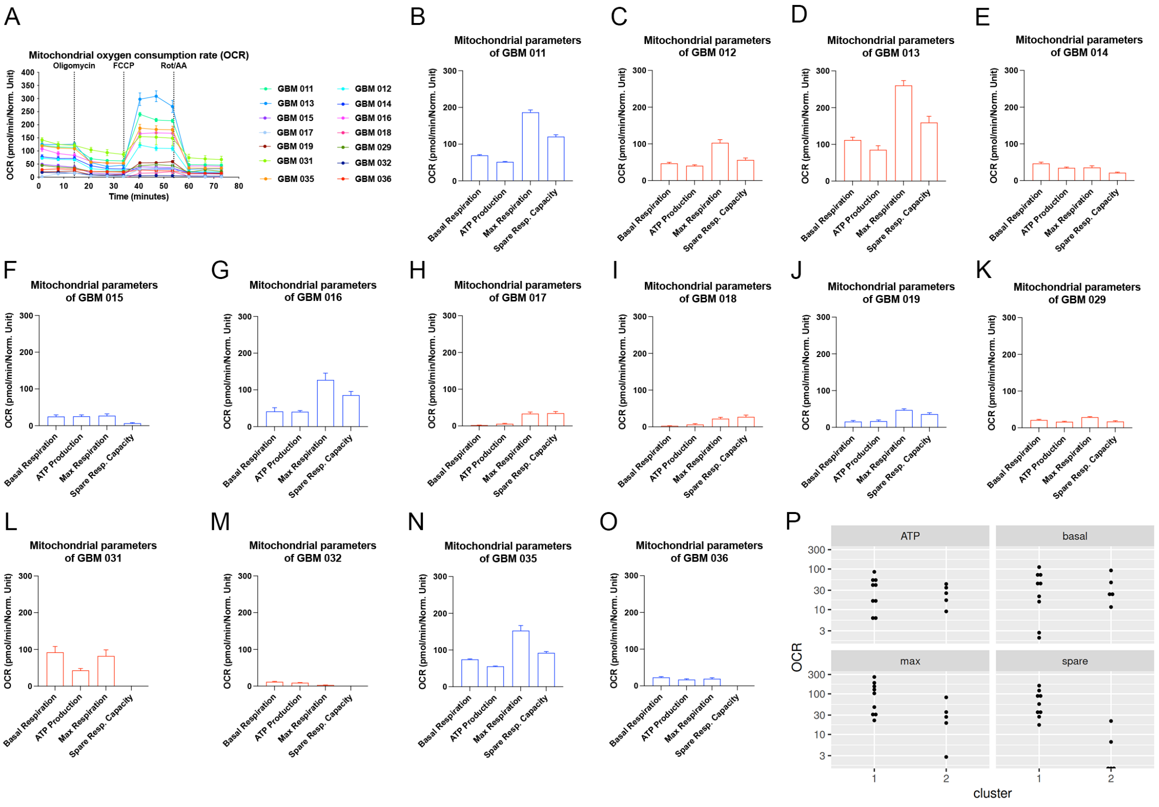

Supplementary Figure 1: Mitochondrial respiration in patient-derived glioblastoma cells after one week *ex vivo*. (A) Normalized mean oxygen consumption rate (OCR) plots from the Mitochondrial Stress Test performed on primary glioblastoma cells using the Seahorse XFe96 Bioanalyzer. OCR was measured in real-time under basal conditions and following sequential injections of oligomycin, FCCP, and rotenone/antimycin A. (B-O) Quantitative analysis of key mitochondrial parameters across individual glioblastoma samples, including basal respiration, ATP production-linked respiration, maximal respiration, and spare respiratory capacity. Bar graphs representing samples derived from female patients are shown in red, whereas bar graphs from male patients are outlined in blue. (P) OCR ranges in the four measured categories in the two clusters (1 and 2). Log-scaled vertical axis

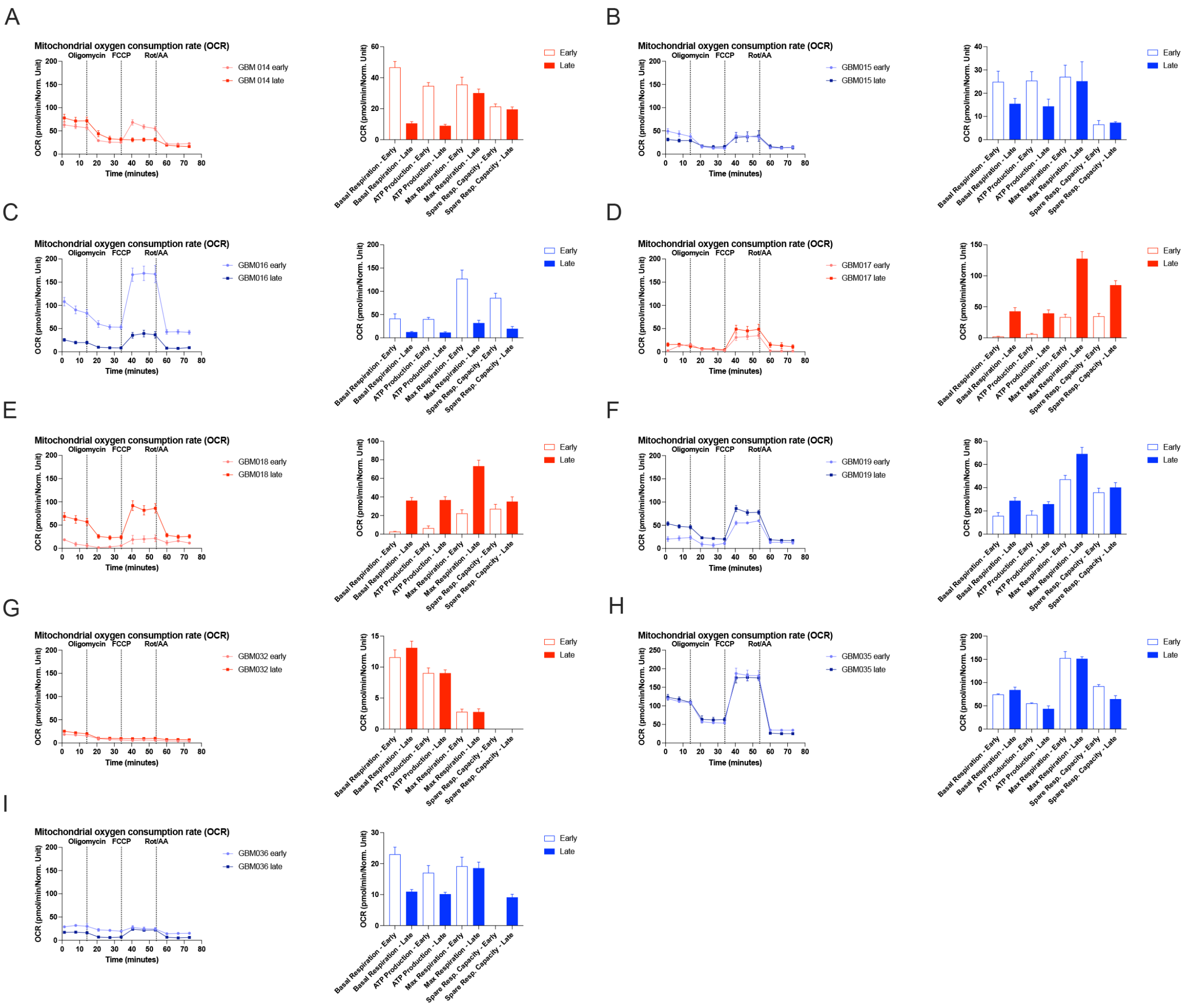

Supplementary Figure 2: Longitudinal analysis of mitochondrial function in patient-derived glioblastoma cultures. (A-I) Oxygen consumption rate (OCR) and mitochondrial parameters of glioblastoma samples that remained viable for longitudinal assessment at one week *ex vivo* and five weeks *ex vivo*. Mitochondrial function was assessed using the Seahorse XFe96 Bioanalyzer, measuring key parameters including basal respiration, ATP production-linked respiration, maximal respiration, and spare respiratory capacity. The comparison of early and late time points reveals heterogeneous adaptation patterns among individual patient-derived glioblastoma cultures, with some samples exhibiting increased mitochondrial activity over time, while others show a decline. No uniform trend was observed across samples, highlighting patient-specific metabolic plasticity. Bars graphs representing samples from female patients are shown in red, whereas bar graphs from male patients are depicted in blue. Each sample was measured in triplicate. Data represent mean ± SEM.

A

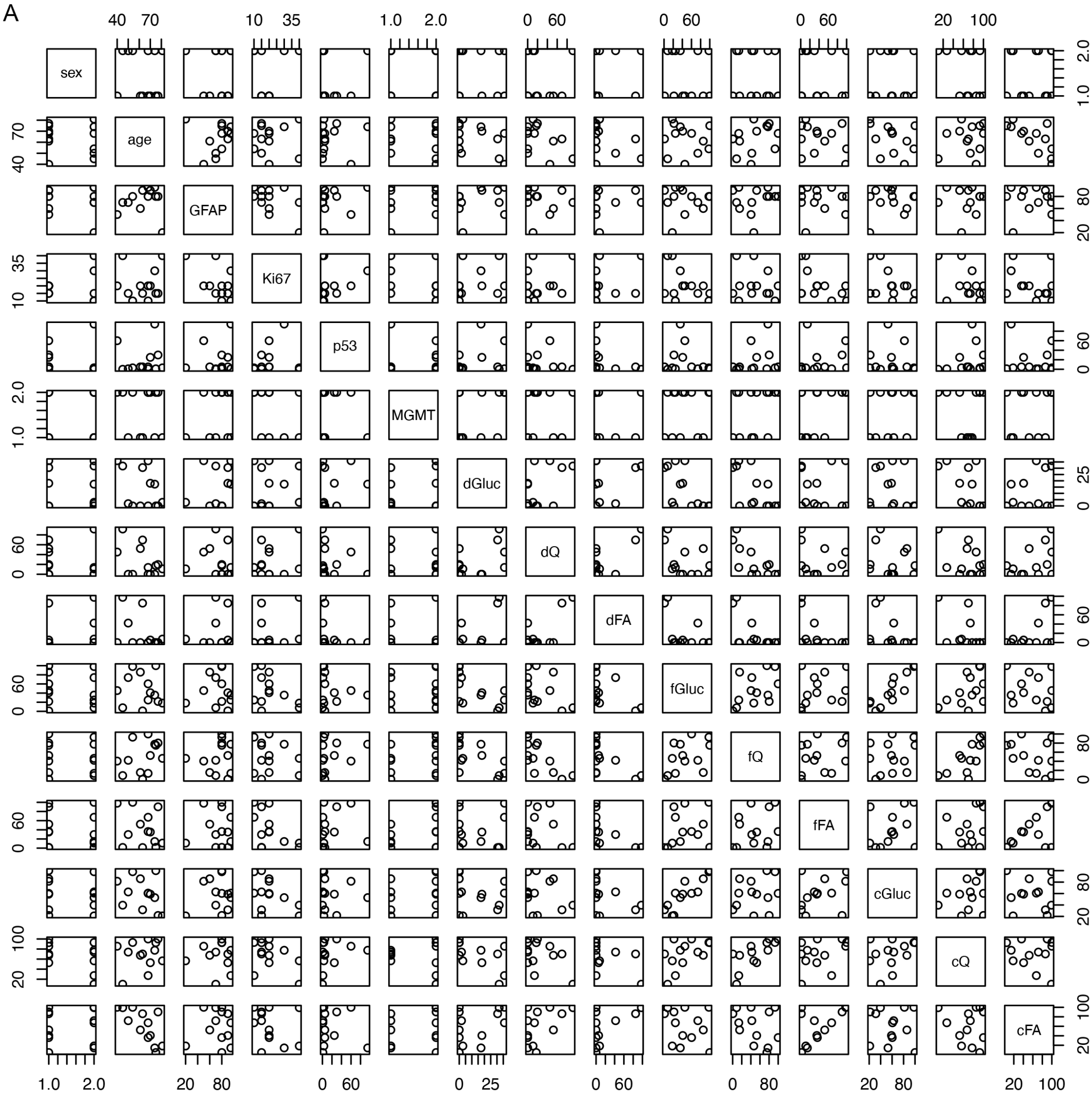

Supplementary Figure 3: (A) An overview of pairwise relationships. For each of the 13 samples (dots), each pair of 12 quantities is plotted (rows and columns). Binary variables were transformed to values 1 and 2, respectively.

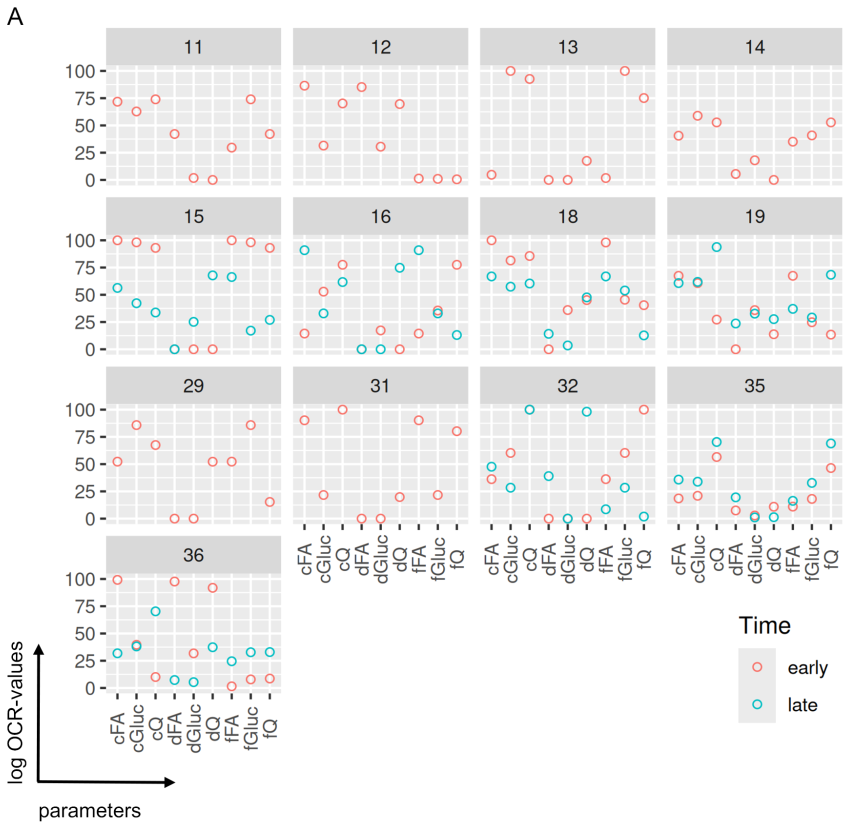

Supplementary Figure 4: (A) Means of Fuel Flex Test data per sample and category for early (red) and late (green) measurements. Oxygen consumption rates (OCR) were measured under basal conditions and following inhibition of glucose oxidation (UK5099), glutamine metabolism (BPTES), and fatty acid β-oxidation (Etomoxir). Dependency (d), capacity (c) and flexibility (f) to oxidize glucose (Gluc), glutamine (Q) and fatty acids (FA) was measured. Each sample was measured in triplicate.

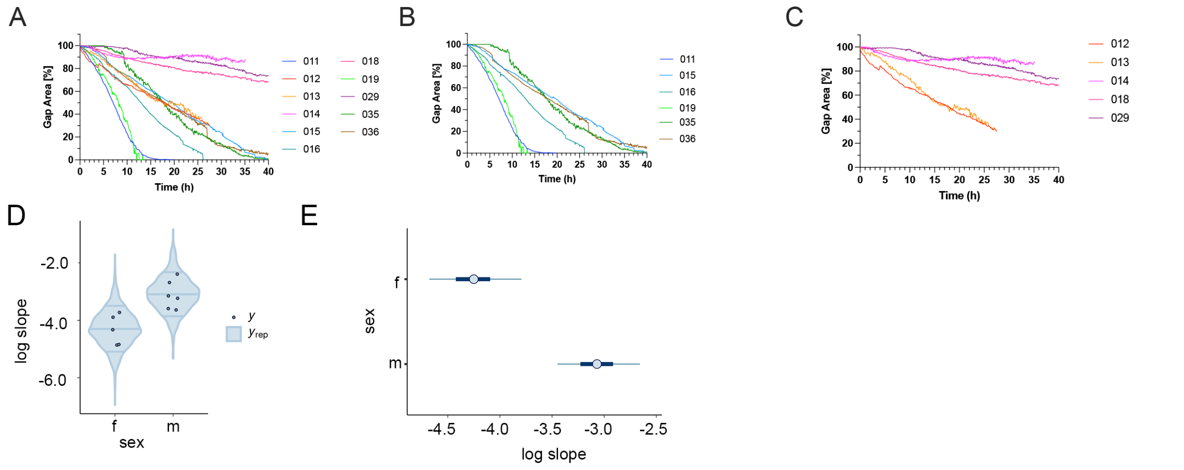

Supplementary Figure 5: Migration dynamics of patient-derived glioblastoma cells at one week *ex vivo*. (A) Time-lapse wound healing assay illustrating the gap closure behavior of individual glioblastoma cultures over 35 hours. Fast-migrating cultures such as GBM011 and GBM019 closed the wound area within 12–15 hours, whereas slower-migrating cultures (e.g., GBM014, GBM018, GBM029) did not achieve complete closure even after 35 hours. (B-C) Stratification of migration kinetics by patient sex revealed significantly faster migration in male-derived glioblastoma cultures (B) compared to female-derived cultures (C). (D) Posterior predictive check (PPC) of Bayesian model for gap closure slopes. Axes indicate sex (horizontal) and log of slope (vertical). Empirical values (dots) are well represented by density of gap closure slopes generated by the model (violins). (E) Marginal posterior of mean gap closure slope in females (top) and males (bottom). Horizontal axis is the log of the slope. Thin uncertainty bars cover 90% of posterior, thick bars 50% of posterior. These results highlight both the inter-patient heterogeneity and sex-dependent differences in glioblastoma cell motility.

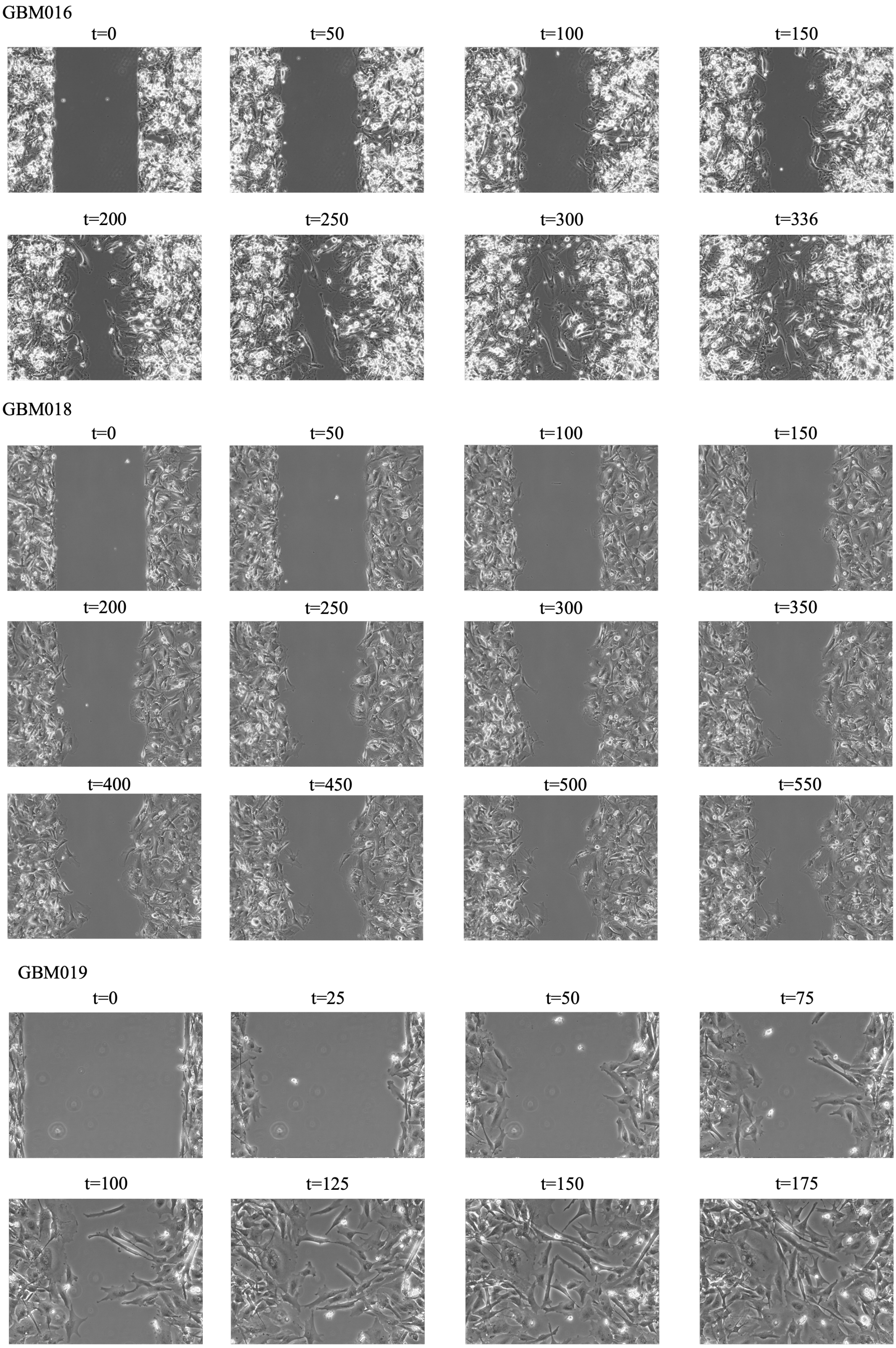

Supplementary Figure 6: Representative time-lapse frames illustrating migratory behavior of patient-derived glioblastoma cultures. Representative phase-contrast images from wound healing assays of GBM016, GBM018, and GBM019 are shown at indicated time points. Cells were imaged every 5 minutes, with t corresponding to the frame number (t = 0 represents the initial time point immediately after insert removal; e.g., t = 50 corresponds to 250 minutes). The selected frames illustrate progressive gap closure and directed cell movement into the wound area. Notably, individual cells can be observed detaching from the cell layer and actively migrating into the gap, supporting that wound closure is primarily driven by cell motility rather than proliferation under these conditions.
